# Importance of Providing Additional Choices in Relation to Pupils' Happiness, Mastery, Well-Being, Contentment, and Level of Physical Activity in Physical Education

**DOI:** 10.3389/fspor.2021.599953

**Published:** 2021-04-15

**Authors:** Stian Oldervik, Pål Lagestad

**Affiliations:** Department of Teaching and Art, Nord University, Levanger, Norway

**Keywords:** autonomy, physical education, activity level, happiness, mastery, well-being, contentment

## Abstract

Previous research points to the importance of providing support to autonomy in PE, because it has a particularly positive effect on motivation in PE. However, previous research has not examined the association between autonomy and the variables; happiness, mastery, well-being, contentment and activity level in PE. This study examined how increased self-determination affects happiness, mastery, well-being, contentment and activity level in PE. The study is an intervention (cross-over study) with one control group (one class) and two intervention groups (two classes), using questionnaires and accelerometers among 88 tenth graders (41 boys and 47 girls). The three classes included approximately the same number of boys and girls. The intervention groups included, respectively, 30 and 29 pupils in each class, and 29 pupils' in the control group. The pupils' experiences of happiness, mastery, well-being, contentment was measured three times–after a month with, respectively, ordinary PE, teacher-directed PE, and self-organized PE (autonomy), and the activity levels (accelerometer) was measured during the 24 lessons that took part in the period of teacher-directed PE and self-organized PE. Factor analysis, repeated measures ANOVA (mixed method ANOVA design) and paired sample *t*-tests with Bonferroni correction were performed, in order to look at differences in happiness, mastery, well-being, contentment and activity level during periods of; self-determination, teacher-directed PE and ordinary PE. The results show that self-determination in PE gives the pupil a significant increased experience of happiness, well-being and contentment, and also a higher activity level in PE. The results indicate that increased self-determination in PE can positively affect lower secondary school pupils' happiness, well-being, contentment and activity level in PE, and that teachers should strive to encourage self-determination among pupils in PE. Further research should be based on intervention studies studying self-determination over longer continuous period, in classes with both older and younger pupils.

## Introduction

Physical education (PE) at school provides structured and regular physical activity (PA) for almost every child around the world (Fairclough and Stratton, 2005a; Long et al., 2013; Chen et al., 2014; Calahorro-Cañada et al., 2017), and is an important arena–taking the decline in PA during adolescence into account (Bélanger et al., 2009; Kolle et al., 2012; Lagestad et al., 2018). PE can help to make children and young people more physically active, helping to bring them the health benefits of this (Loprinzi et al., 2012; Meyer et al., 2013). Children and adolescents who engage in moderate-to-vigorous PA (MVPA) are at lower risk of developing chronic health issues (Janssen and Leblanc, 2010). An active lifestyle and lifelong pleasure in movement are major aims of PE in Norwegian schools (Norwegian Directorate of Education Training, 2019), and happiness, mastery, well-being, contentment and PA may be important indicators of the achievement of these aims. Hassandra et al. (2003), showed that PE can be important in helping to promote PA into adulthood. A study by Kalajas-Tilga et al. (2020) concluded that to enhance adolescents' daily MVPA, special focus should be put on increasing their intrinsic motivation toward physical education. Delextrat et al. (2020) concluded that activity type could be associated with the intensity of PA in PE.

Self-determination in relation to choosing activities in PE is one of the core values in the new curriculum in Norway (Norwegian Directorate of Education Training, 2019). Earlier research has shown that self-determination has a particularly positive effect on motivation in PE (Deci and Ryan, 1985, 2000; Ryan and Deci, 2007), in which the psychological need for autonomy, competence and belonging are seen as necessary in order to increase inner motivation (Ryan and Deci, 2007). Self-determination theory suggests that the level of activity, happiness, feelings of competence and mastery, well-being and contentment in PE, can all be increased by self-determination (Deci and Ryan, 1985, 2000). Inner motivation comes from within, leading to the kind of behaviors we want to pursue. Outer motivation on the other hand, comes from an external source. Inner motivation is especially important for children and young people in terms of a lifelong delight in movement and is closely connected to learning (Deci and Ryan, 1985).

Pupils will benefit from teachers' adoption of an autonomy supportive teaching style, as pointed out by Cheon and Reeve (2013). A study showed that pupils with more autonomous motivational profiles, reported being more active at secondary school and in early adulthood (Haerens et al., 2010). In their study, including secondary school pupils (13 years of age) reporting on their PE teachers, Haerens et al. (2018) found that in relation to four clustered motivating profiles, the high-autonomy support group showed the most optimal pattern of outcomes (e.g., need satisfaction, autonomous motivation). Another study showed that all pupils–independent of their motivational regulations, were more engaged and showed less oppositional defiance when they interacted with an autonomy-supportive teacher, instead of a controlling teacher during PE (De Meyer et al., 2016). Also a study by Van den Berghe et al. (2015) found that pupils behavioral and emotional engagement was positively related to need support in PE. Interventions have also been successful in increasing autonomy support among teachers (Tessier et al., 2010; Cheon et al., 2012; Aelterman et al., 2014; Cheon and Reeve, 2015; Ulstad et al., 2018).

Several intervention studies have looked at autonomy in PE from a pupil perspective–indicating that autonomy can positively affect pupils' happiness, mastery, well-being and contentment. Two studies found a positive association between self-determination in PE and PA and motivation in PE (Lonsdale et al., 2009, 2013). found that the sense of autonomy was raised if the pupils were able to make choices. Several other studies also indicate that self-determined instruction is positively reflected in the pupils' motivation (Prusak et al., 2004; Ntoumanis, 2005; Ward et al., 2008; How et al., 2013). Ward et al. (2008), Prusak et al. (2004); Lonsdale et al. (2009, 2013), and How et al. (2013), all measured pupils' motivation. However, these studies look primarily at pupils' inner motivation. No experimental study examine how self-determination affects pupils experiences of happiness, mastery, well-being or contentment in a study. However, studies have examined some of these variables. The findings of Ntoumanis (2005) suggest that if teachers manage to support pupils' basic psychological needs, they positively impact pupils' well-being. Furthermore, the study of (Lagestad, 2017), indicate that autonomy can positively affect pupils' happiness, mastery, well-being and contentment, but they did not measure the relationship between these variables, and providing additional choices.

Although a number of studies indicate that increased self-determination in PE can positively affect happiness, mastery, well-being, contentment and activity level, earlier research has not studied these connections by means of interventions taking in these variables, using pre-post designs. Some of these studies do not have a pupil perspective, and the activity level is not measured with an accelerometer–which is the preferred method (Kolle et al., 2012). Fairclough and Stratton (2005b) point out that few studies have investigated psychological factors and activity level, whilst referring to the relationship between inner motivation, effort, and happiness. They suggest that it can be expected that pupils' enjoyment, activity level and motivation will increase in unison, and that one can also propose that mastery, well-being and contentment will be positively affected. Intervention and experimental studies has been highlighted as important in the future research on self-determination in PE (Van den Berghe et al., 2014). Taking other research into account (Chen et al., 2014; Gao et al., 2015), we will argue that happiness, mastery and well-being during PA in PE, will help adolescents to apply more PA into their daily regime, and thereby positively affect PA in a lifelong perspective–a main aim of PE in Norwegian schools (Norwegian Directorate of Education Training, 2019).

From a pupils' perspective the present study will explore the extent to which increased autonomy affects pupils' feelings and behavior PE. The question that this study addresses is the following: To what extent will self-selected activities in PE affect high school pupils' happiness, mastery, well-being, contentment and level of activity in the subject of PE, compared to ordinary and teacher-directed instruction? Our hypothesis is that increased autonomy will positively affect these variables, and that a teacher-directed instruction style (with no self-selected activities in PE at all) will negatively affect these variables.

## Method

### Participants and Design

In order to address this research question, an intervention crossover study (a longitudinal study in which the participants receive three different treatments) were carried out, comprising questionnaires, accelerometer measurements and observations. Based on power calculations (Cohen, 1988) related to a previous study (Cheon et al., 2012), with standards deviation (SD = 0.23) and expected differences between groups (Δ = 0.39, *α* = 0.05, *β* = 0.8), at least 53 participants had to be included in the study. On the basis of a stratified selection, a local authority high school in a mid-Norwegian town was chosen, and three 10th grade classes with, together, 88 pupils (age 15–16) were selected. The intervention groups included, respectively, 30 and 29 pupils in each class, and 29 pupils in the control group. These three classes shared the same PE teacher, which was a requirement for participation in the study. Furthermore, the PE teacher was both qualified and willing to do the experiment. A random selection was undertaken to establish which classes would be a control group and which intervention groups 1 and 2. The research project and the ethics of the study was approved by the Norwegian Centre for Research Data (NSD), and the headteacher, the teacher and all the participating pupils and their parents signed an informed consent.

### Data Collection

**Figure 1:**
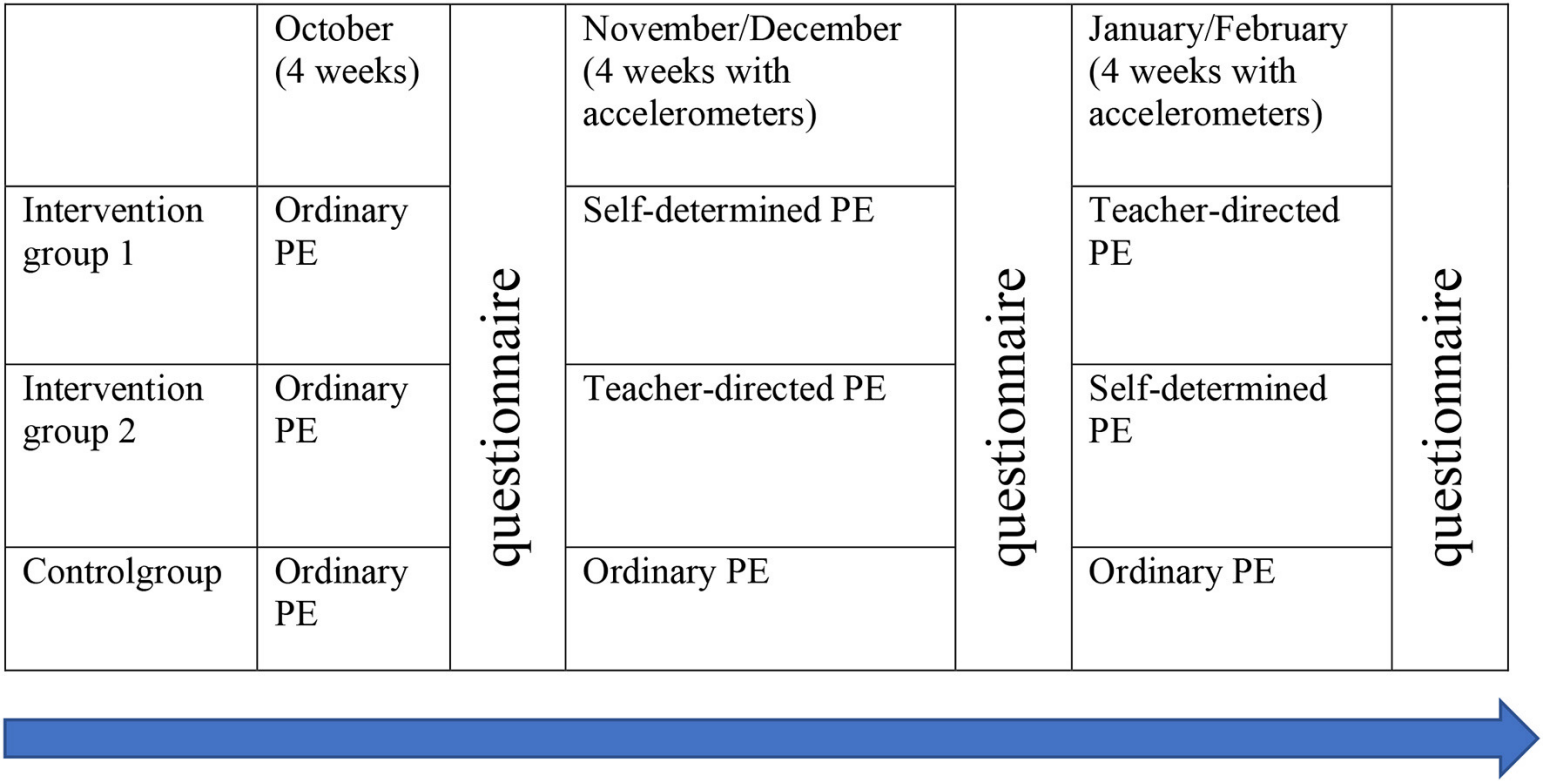
The organization of the data collection.

Data collection took place between November 2018 and February 2019. The first period (1 month) all three groups had ordinary instructions, and the pupils completed a questionnaire concerning their understanding of happiness, mastery, well-being and contentment in relation to PE at the end of October/beginning of November 2018 (Figure 1). In period 2 (November/December 2019) the three classes had different PE instruction in 4 PE lessons (each of 90 min duration) per class during a 30-day period (one lesson per week in line with the Norwegian curriculum). Intervention group 1 carried out self-determined PE, while intervention group 2 had teacher-directed PE. This regime took place in a 1-month continuous period (4 lessons of 90 min each). During these 4 lessons all three groups wore an accelerometer to measure their PA. The pupils wore accelerometer only during the PE lessons. In period 3 (January/February 2020) the intervention groups “crossed over” (intervention group 1 carried out teacher-directed PE, while intervention group 2 had self-determined PE) with the same test protocol, answering questionnaires at the end of the month. The control group conducted the same test regime but had ordinary instruction all 3 months. The teacher self-determined PE was organized by letting the pupils chose their own activity in a 1-month continuous period (4 lessons of 90 min each). The teacher-directed PE was designed in the way that the teacher was told to organize all lessons and to have a clear “teacher leading style,” as described in Brattenborg and Engebretsen (2007), during a 1-month continuous period (4 lessons of 90 min each). Based on our hypothesis, the PE teacher was instructed to have a teacher-directed PE style. It was essential that he should offer the pupils no additional choices at all, where the pupils were told to just follow the instructions from the teacher. The teacher were told to use a “follow me” instruction, telling the pupils exactly what to do in each activity. The teacher-directed style were in this project defined as a strategy were the teacher had control over which activities that should be conducted, and how the activities he had planned for the PE lessons should be organized. Because we also wanted to study the effect of ordinary teaching, the PE teacher were told to organize the ordinary instruction during a 1 month continuous period (4 lessons of 90 min each), using the teaching style that the teacher used in his ordinary PE lessons. Teacher and pupils were also observed during these 12 lessons, and after the 30 days the same questionnaire was answered again. During January and February, there was a further 30-day period of differing instruction in which the control group again had ordinary PE, but where intervention group 1 had teacher-led activities and intervention group 2 had self-determined PA (crossover study). Also during these 30 days, the pupils wore an accelerometer to measure their activity level, and answered the same questionnaire at the end of the period.

### Measures

The questions used to assess happiness, mastery, well-being and contentment had been specifically developed for PE and PA research, and had been used earlier in the REPAC project (Säfvenbom et al., 2018). Whilst these questions are closely connected to these variables, they also connect to self-determination as a theoretical framework based on; basic psychological needs (Vlachopoulos and Michailidou, 2006), motivation (Guay et al., 2000), belonging (Anderson-Butcher and Conroy, 2002) and enthusiasm (Lagestad et al., 2019). Each question/statement offered alternative answers on a 1-7 Likert scale. Several questions (mostly statements)–most of the questions with a high face validity according to the dependent variables; happiness, mastery, well-being and contentment, was included in each variable. This strategy to create the dependent variables was based upon theoretical constructions, using factor analysis to measure the reliability. Happiness included the five questions: “Because PE is fun and exciting,” “Because it feels good to have PE,” “I always looking forward to PE,” “I want to have PE,” and “I am always happy in PE.” Mastery included the four questions: “I feel I have a great progress according to the main aim of PE,” “I feel I am doing the exercises in PE the right way,” “I feel I am doing the activities in PE very well,” “I feel I can master the PE tasks.” Well-being included the six questions: “I feel very convenient together with the other pupils in PE,” “Because I feel PE is nice,” “I feel I am an important person in PE,” “I wish I was not a part of the group in PE [the values in this question were changed],” “I feel like being accepted in PE,” and “I enjoy having PE.” Contentment included the four questions: “What do you feel about the PE lessons?,” “What do you feel about the organization of the PE lessons?,” “I feel strongly that PE fits me,” and “The PE lessons definitely reflects how I wish PE to be.”

Observations were made in order to ensure that the different groups carried out the PE in accord with the intention for each group in each period, the quality of the accelerometer measurements, as well as gathering observational data which could help the quantitative analysis around happiness, mastery, well-being, contentment and PA. The same observer were present in all 24 lessons during the data collection period. This observation ensured that the procedures was followed in each class at every time. The accelerometers where given out and back at the exact same place each time, the absence where documented in the same way, and during the observation, all activities that was conducted at each lesson was documented. As pointed out by Haerens et al. (2013), in studies related to changes in need-supportive teaching practices after exposure to an intervention, observations may benefit the study greatly. ActiGraph GTM1 (ActiGraph, Fort Walton Beach, Florida) accelerometers were used to measure the young peoples' moderate and vigorous PA (MVPA) during PE. These were tested in advance of the project, being used continuously for a week. Each of the 88 pupils used the same accelerometer in all the lessons during the time measurements were being taken, and the data gathered was uploaded to Actilife software and processed every 14 days. The raw data files were measured at 5 second intervals (epochs). Periods of time where data were missing were defined, in conformity with Kolle et al. (2012), as a continuous period of 20 min or more where the accelerometer had measured no counts. The limit for moderate intensity was set at 2,000 counts, according to the limits set in other studies (Kolle et al., 2012; Meyer et al., 2013; Andersen, 2017). All the pupils included in the study returned valid questionnaires and accelerometer data.

### Statistical Analysis

Factor analysis was used to look at whether the four to six questions included in the different indexes belonged together in meaningful clusters, in order to confirm a pre-proposed connection between different variables (confirmatory approach). The factor analysis showed that all the different indexes had an eigenvalue well over 1 (from 2.9 to 4.0), that all the questions in all the different indexes had a high common covariance (between 0.7 and 0.9) and that the total variance shown by the questions included in the different indexes was high (between 0.70 and 0.80). Furthermore, there were no multicollinearity between the questions included in the indexes.

A mixed method ANOVA design was used to study changes through time (Gray and Kinnear, 2012) in happiness, mastery, well-being, contentment and possibility to choose, as well as revealing the differences between the three groups, using *post-hoc* tests with Bonferroni corrections. Effect size was reported with the help of $\eta_{\text{p}}^{2}$ (partial eta-squared), where 0.01 < *η*^2^ < 0.06 indicates a small effect, 0.06 < *η*^2^ < 0.14a medium effect, and *η*^2^ > 0.14a large effect (Cohen, 1988). *T*-tests with Bonferroni corrections were used to show differences between the groups according to periods of different teacher style (self-determined PE, ordinary PE and teacher-directed PE), where mastery, happiness, possibility of making choices, well-being, contentment and activity level were concerned. SPSS data analytic software (version 25.0: IBM, Armonk, NY, USA) was used to analyse the data. The observation notes were used to expand and complement the quantitative data, as well as possibly supporting the analytic findings.

## Results

**Figure 2:**
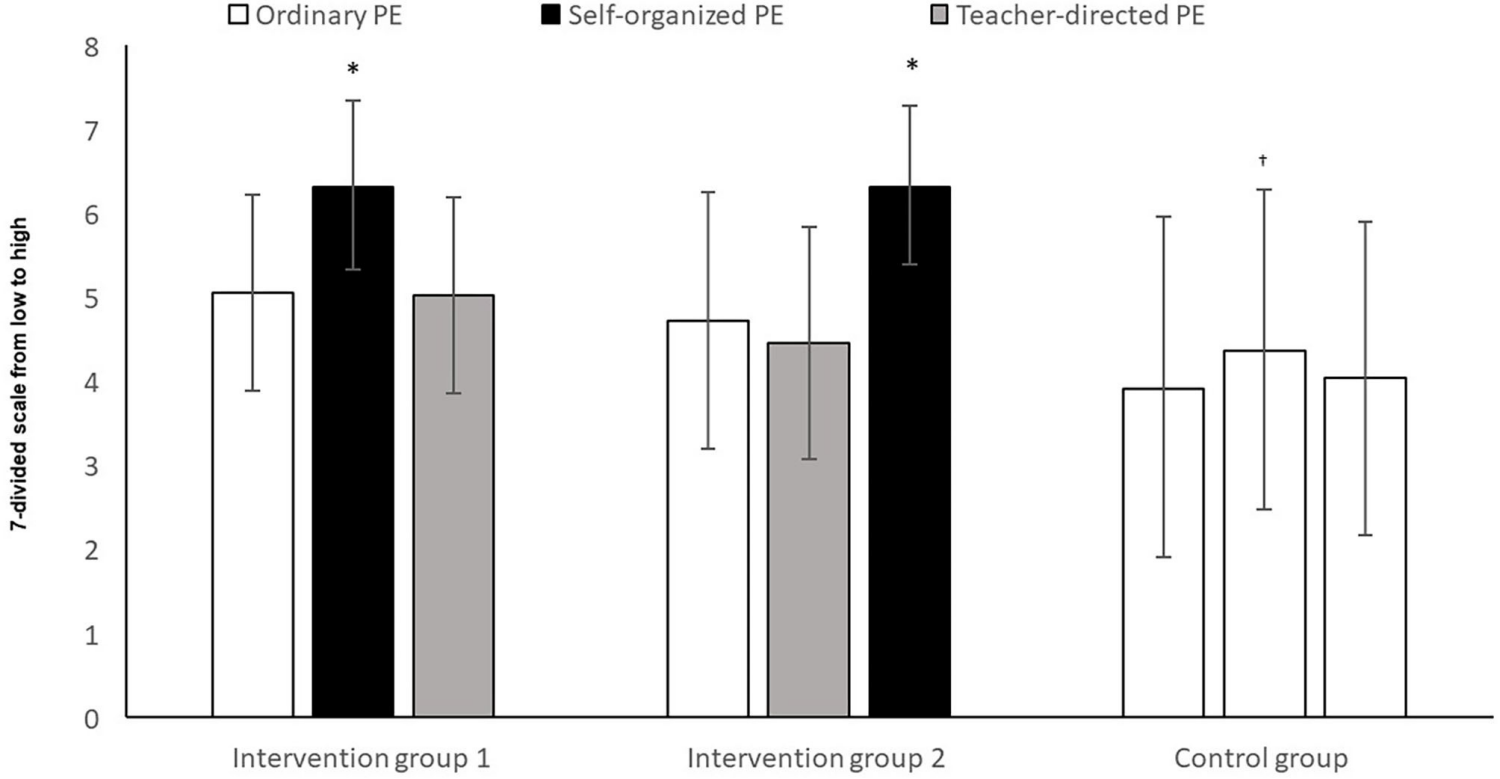
Perception of having choice among the pupils after three periods of ordinary PE, teacher-led PE and self-determined. *Significantly greater perception of choice in self-determined PE, compared to ordinary and teacher-led PE (*p* < 0.05). ^†^Significantly lower perception of choice in the control group, compared to intervention groups 1 and 2 (*p* < 0.05).

The analysis showed a significant change in the groups, over time (Figure 2), in the perception of having choice (*F*_2,166_ = 5.65, *p* = 0.008, *η*^2^ = 0.06). There was also a significant difference in perception of choice between the groups (*F*_2,83_ = 11.86, *p* = 0.000, *η*^2^ = 0.22), together with an interaction between time and group (*F*_4,166_ = 11.87, *p* = 0.000, *η*^2^ = 0.22). *Post-hoc* tests with Bonferroni corrections showed that both intervention group 1 and intervention group 2 reported a significantly higher perception of having choice, than the control group (mean difference = 1.35, 95% CI = 0.6, 2.1, *p* = 0.000 and mean difference = 1.06, 95% CI = 0.4, 1.8, *p* = 0.001), but there was no significant difference between the two intervention groups (*p* = 0.968).

Intervention group 1 experienced a significantly greater degree of choice in self-determined PE, compared to both ordinary PE (*t* = −4.45, *p* < 0.05) and teacher-directed PE (*t* = −6.18, *p* < 0.05). There was, however, no significant difference between ordinary PE and teacher-directed PE in intervention group 1 (*t* = 0.14, *p* > 0.05).

Intervention group 2 experienced a significantly greater degree of choice in self-determined PE compared to both ordinary PE (*t* = −5.11, *p* < 0.05) and teacher-directed PE (*t* = −6.18, *p* < 0.05). There was, however, no significant difference between ordinary PE and teacher-directed PE in intervention group 2 (*t* = 1.00, *p* > 0.05).

There were no significant differences in the control group in respect of perception of opportunities for choice between T1 and T2 (*t* = −1.14, *p* > 0.05), T2 and T3 (*t* = 0.94, *p* > 0.05), nor between T1 and T3 (*t* = −0.22, *p* > 0.05).

**Figure 3:**
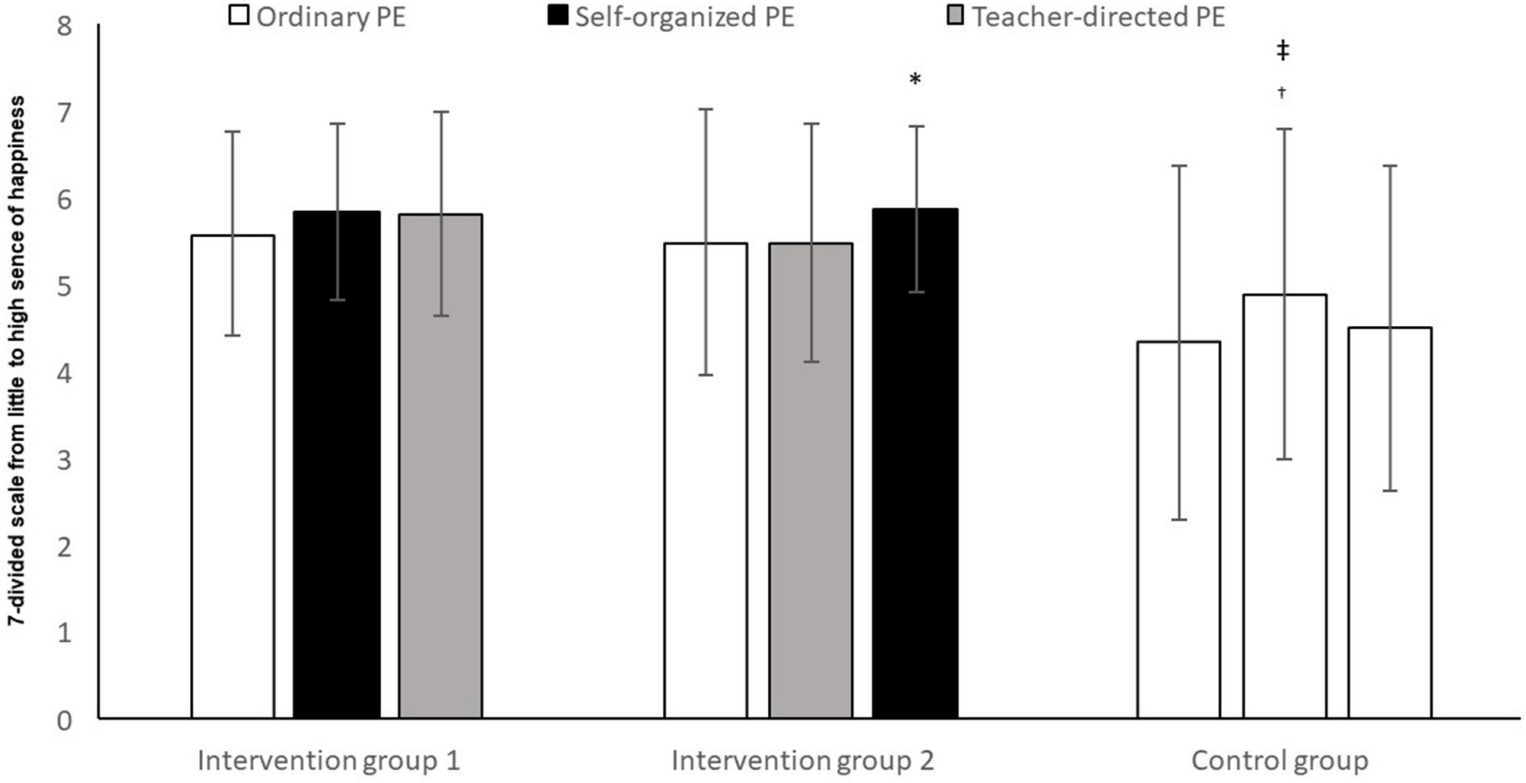
Perception of happiness among pupils in ordinary PE, teacher-directed PE and self-determined PE after 3 time periods. *Significantly higher perception of happiness in self-determined PE compared to teacher-directed PE (*p* < 0.05). ^†^Significantly lower perception of happiness in the control group, compared to intervention groups 1 and 2 (*p* < 0.05). ^‡^ Significantly higher perception of happiness in the control group in T2 compared to T1 (*p* < 0.05).

The analysis showed no significant change (Figure 3), over time, between the groups in their perception of happiness (*F*_2,148_ = 2.35, *p* = 0.099, *η*^2^ = 0.03). There was, however, a significant difference between the groups in their perception of happiness (*F*_2,74_ = 8.31, *p* = 0.001, *η*^2^ = 0.18), but no interaction between time and group (*F*_4,148_ = 2.16, *p* = 0.076, *η*^2^ = 0.06). *Post-hoc* tests with Bonferroni corrections showed that both intervention group 1 and intervention group 2 reported significantly more perception of happiness than the control group (mean difference = −7.56, 95% CI = −12.4, −2.7, *p* = 0.001 and mean difference = −5.86, 95% CI = −10.4, −1.3, *p* = 0.007), but there was no significant difference between intervention group 1 and intervention group 2 (*p* = 1.000).

Intervention group 1 experienced no significantly higher degree of happiness in self-determined PE than in either ordinary PE (*p* > 0.05) or teacher-directed PE (*t* = −0.09, *p* > 0.05). Nor was there any significant difference in intervention group 1 between ordinary PE and teacher-directed PE (*t* = −1.73, *p* > 0.05).

Intervention group 2 experienced a significantly higher degree of happiness in self-determined PE than in teacher-directed PE (*t* = −2.69, *p* < 0.05). They experienced no significantly higher degree of happiness in self-determined PE than in ordinary PE (*t* = −1.86, *p* > 0.05). Nor was there any significant difference between ordinary PE and teacher-directed PE in intervention group 2 (*t* = 0.04, *p* > 0.05).

In the control group, there was a significant difference in their experience of happiness between T1 and T2 (*t* = −2.17, *p* < 0.05). There was, however, no significant difference in their experience of happiness between T2 and T3 (*t* = 1.71, *p* > 0.05), nor between T2 and T3 (*t* = −0.44, *p* > 0.05).

**Figure 4:**
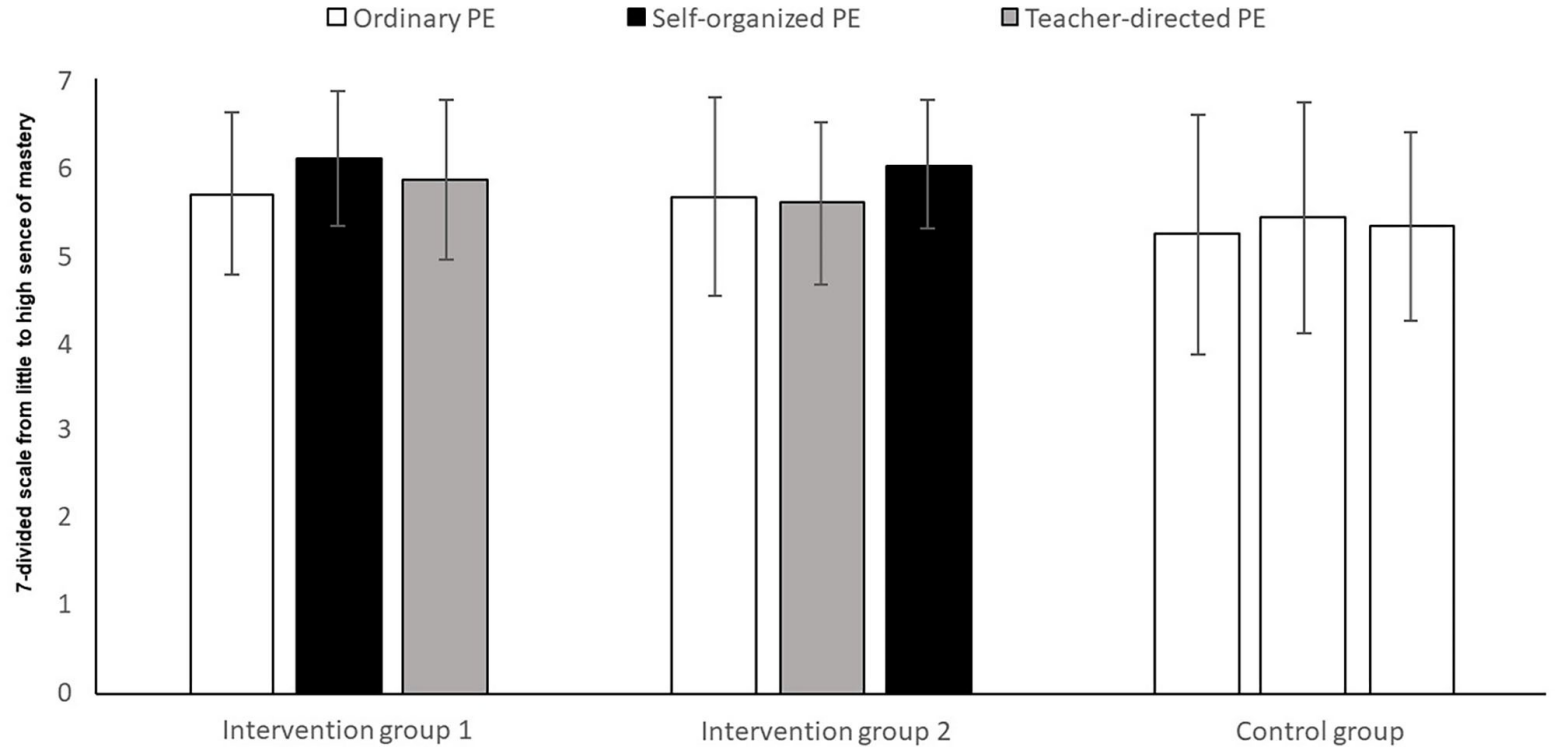
Perception of mastery among pupils after three time periods of ordinary PE, teacher-directed PE, or self-determined PE.

The analysis showed no significant change (Figure 4), over time, in the perception of mastery of the groups (*F*_2,156_ = 2.63, *p* = 0.075, *η*^2^ = 0.03). Nor was there a significant difference between the groups (*F*_2,78_ = 2.76, *p* = 0.066, *η*^2^ = 0.07), nor any interaction between time and group (*F*_4,156_ = 1.85, *p* = 0.123, *η*^2^ = 0.05).

**Figure 5:**
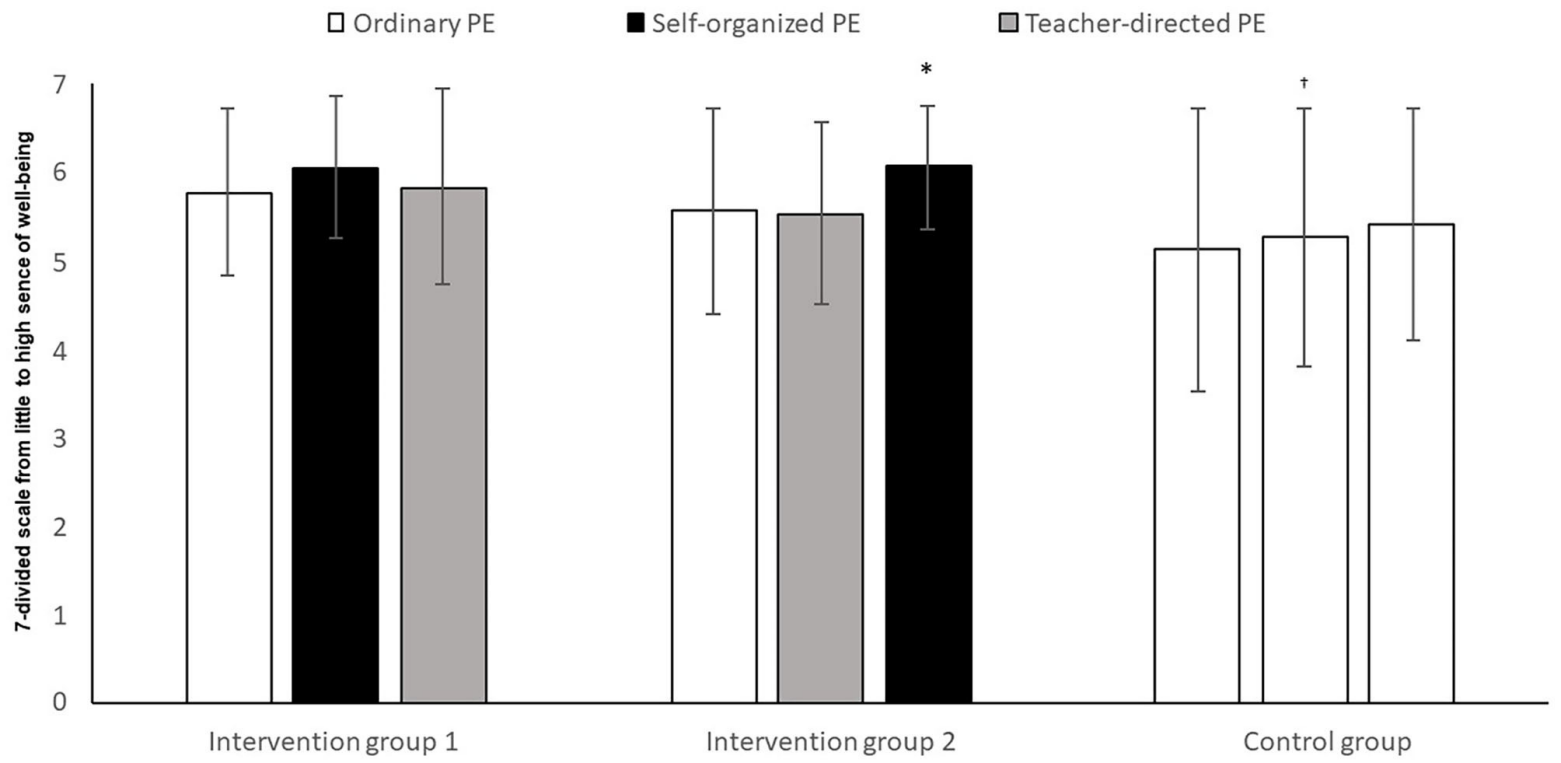
Experience of well-being among pupils after three time periods of ordinary PE, teacher-directed PE, or self-determined PE. *Significantly higher perception of well-being in self-determined PE, compared to ordinary instruction or teacher-directed (*p* < 0.05). ^†^Significantly lower perception of well-being in the control group, compared to intervention groups 1 and 2 (*p* < 0.05).

The analysis showed a significant change in well-being between the groups (Figure 5), over time (*F*_4,156_ = 5.64, *p* = 0.004, *η*^2^ = 0.05). There was also a significant difference in well-being between the groups (*F*_2,74_ = 3.66, *p* = 0.030, *η*^2^ = 0.09). There was, however, no interaction between time and group (*F*_4,148_ = 1.55, *p* = 0.190, *η*^2^ = 0.04). *Post-hoc* tests with Bonferroni corrections showed that intervention group 1 reported significantly more well-being than the control group (mean difference = 4.76, 95% CI = 0.4, 9.1, *p* = 0.009), but the analysis found no significant differences between intervention group 2 and the control group (*p* = 0.241) or between intervention group 1 and 2 (*p* = 0.878).

Intervention group 1 experienced no significantly higher level of well-being in self-determined PE than in ordinary PE (*t* = −1.51, *p* > 0.05), or in teacher-directed PE (*t* = 0.75, *p* > 0.05). Neither, in intervention group 1, was there any significant difference between ordinary PE and teacher-directed PE (*t* = −1.41, *p* > 0.05).

There were no significant differences in the control group where perception of well-being was concerned between T1 and T2 (*t* = −0.79, *p* > 0.05), T2 and T3 (*t* = −0.84, *p* > 0.05), nor between T1 and T3 (*t* = −1.46, *p* > 0.05).

**Figure 6:**
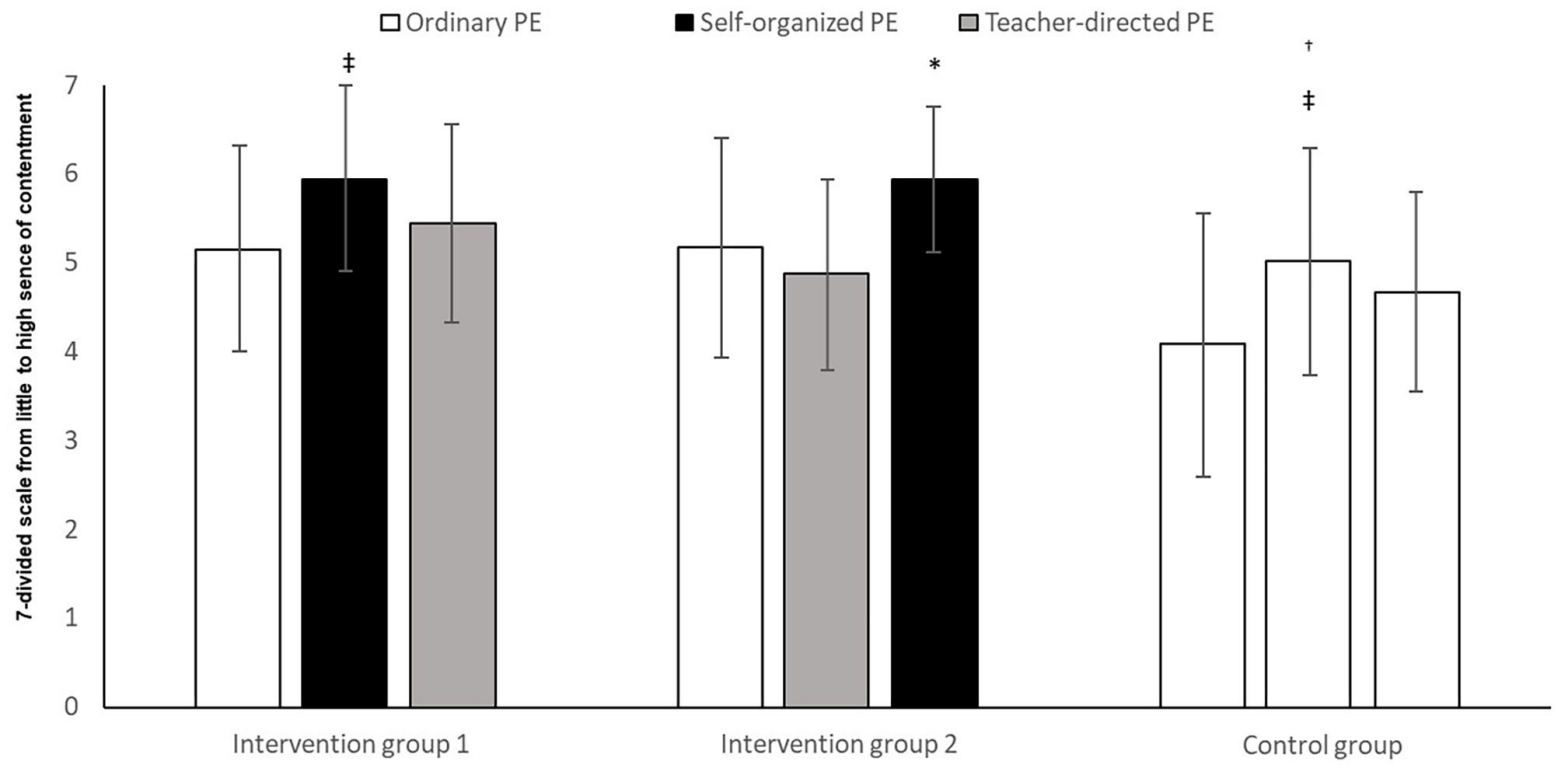
Experience of contentment among pupils after three time periods of ordinary PE, teacher-directed PE, or self-determined PE. *Significantly higher experience of contentment with self-determined PE, compared with ordinary PE and teacher-directed PE (*p* < 0.05). ^‡^Significantly higher experience of contentment in self-determined PE, compared to ordinary PE (*p* < 0.05). ^†^Significantly lower experience of contentment in the control group, compared with intervention groups 1 and 2 (*p* < 0.05). ^‡^Significantly higher experience of contentment in T2, compared to T1 (*p* < 0.05).

The analysis showed a significant change (Figure 6), over time, in the experience of contentment between the groups (*F*_2,158_ = 9.74, *p* = 0.003, *η*^2^ = 0.11). There was also a significant difference in the experience of contentment between the groups (*F*_2,79_ = 7.46, *p* = 0.001, *η*^2^ = 0.16), and an interaction between time and group (*F*_4,158_ = 9.62, *p* = 0.000, *η*^2^ = 0.20). *Post-hoc* tests with Bonferroni corrections showed that both intervention group 1 and intervention group 2 reported a significantly higher level of contentment than the control group (mean difference = 3.83, 95% CI = 1.3, 6.4, *p* = 0.001 and mean difference = 2.78, 95% CI = 0.3, 5.2, *p* = 0.020), but there was no significant difference between intervention group 1 and intervention group 2 (*p* = 0.920).

Intervention group 1 experienced a significantly higher level of contentment in self-determined PE than those in ordinary PE (*t* = −3.50, *p* < 0.05). They did not experience a significantly higher level of contentment than those in teacher-directed PE (*t* = 1.98, *p* > 0.05). Nor were there any significant differences between ordinary PE and teacher-directed PE in intervention group 1 (*t* = −1.36, *p* > 0.05).

Intervention group 2 experienced a significantly higher level of contentment in self-determined PE than those in teacher-directed PE (*t* = −5.19, *p* < 0.05) and ordinary PE (*t* = −3.20, *p* < 0.05). There were, however, no significant differences between ordinary PE and teacher-directed PE in intervention group 2 (*t* = 1.60, *p* > 0.05).

In the control group, there was a significant increase in the perception of contentment between T1 and T2 (*t* = –3.81, *p* < 0.05), and T1-T3 (*t* = –2.47, *p* < 0.05). There was, however, no significant increase between T2 and T3 (*p* > 0.05).

**Figure 7:**
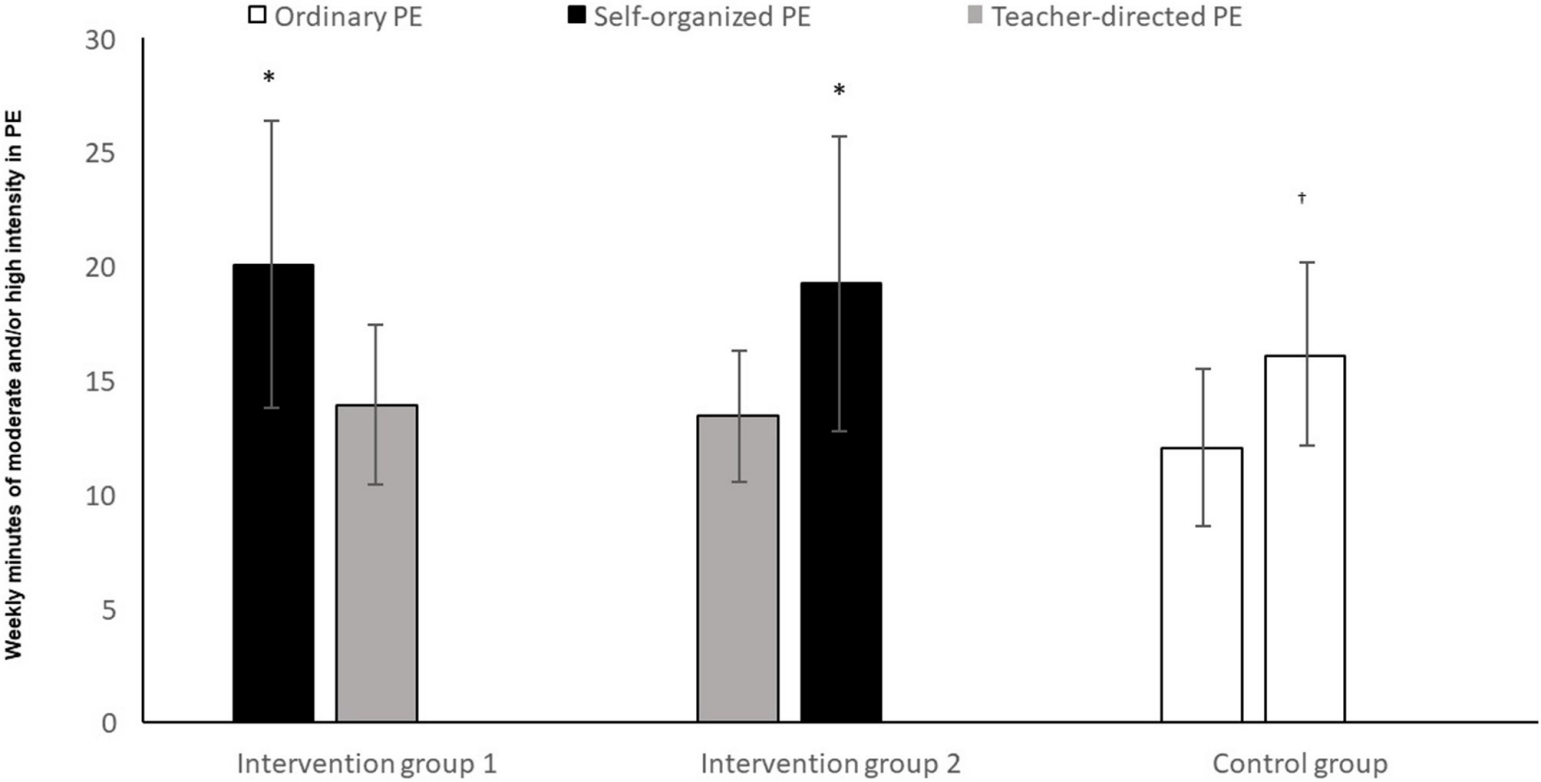
Level of PA among pupils in two time periods of ordinary PE, teacher-directed PE, and self-determined PE. *Significantly higher level of PA with self-determination compared to teacher-directed instruction (*p* < 0.05). ^†^Significantly higher level of activity in T2, compared to T1 (*p* < 0.05).

The analysis showed that both intervention group 1 and intervention group 2 had significantly higher MVPA in self-determined PE (Figure 7) than in teacher-directed PE (*t* = 6.03, *p* < 0.05 and *t* = –5.38, *p* < 0.05). The control group had significantly lower MVPA in period 1, compared to period 2 (*t* = –5.99, *p* < 0.05).

**Figure 8:**
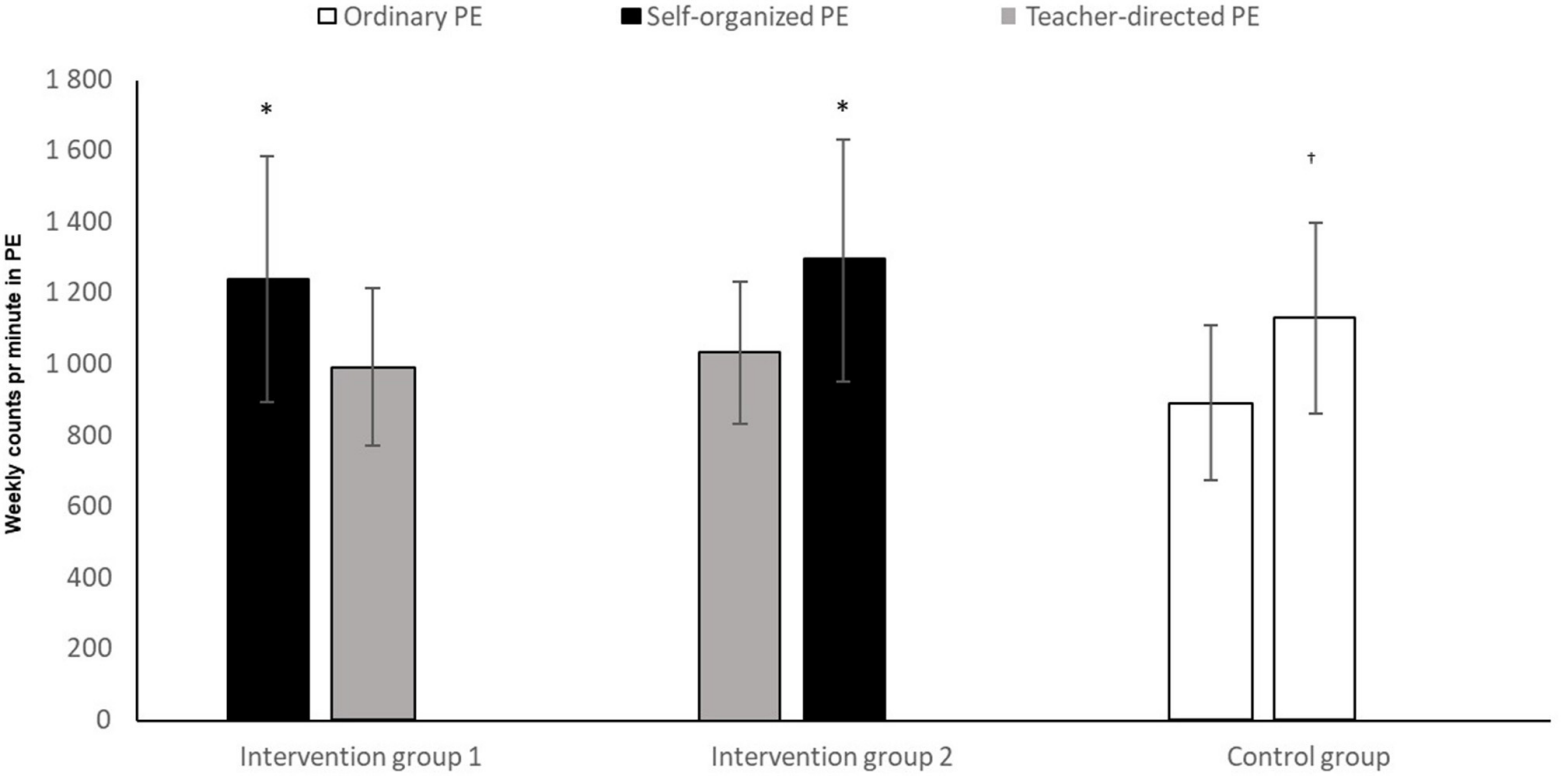
Number of counts per minute among pupils in two time periods of ordinary PE, teacher-directed PE, and self-determined PE. *Significantly higher number of counts per minute with self-determination, compared to teacher-directed instruction (*p* < 0.05). ^†^Significantly higher number of counts per minute in T2, compared to T1 (*p* < 0.05).

The analysis showed that both intervention group 1 and intervention group 2 had significantly higher CPM in self-determined PE (Figure 8) than in teacher-directed PE (*t* = 4.45, *p* < 0.05 and *t* = –4.67, *p* < 0.05). The control group had significantly lower MVPA in period 1, compared to period 2 (*t* = –5.10, *p* < 0.05).

The observations showed that in the teacher-directed PE and ordinary PE the teacher organized and led the activities: Volleyball, strength training, relays, running technique, bandy, obstacle courses, gymnastic, football and capture the flag. In the self-determined PE, the pupils chose more aesthetic activities, such as dancing, gymnastic and martial Arts. Some of the pupils chose strength training/flexibility training. The majority in the intervention group 2 chose volleyball as an activity.

## Discussion

The theoretical basis of this study was to investigate whether a greater degree of self-determination, or autonomy, affected pupils' happiness, mastery, well-being and contentment in PE. The results clearly show that the intervention groups, unlike the control group, experienced significantly greater perception of choice in self-determined PE (autonomy support), in the periods in which they were given it, and that the intervention therefore functioned in keeping with the study's theoretical point of departure (Deci and Ryan, 1985, 2000; Ryan and Deci, 2007). Such a confirmation is important to legitimate our findings in which the experience of co-determination is shown to have a positive influence on happiness, mastery, well-being and contentment in PE. Although not all correlations are significant after strict Bonferroni corrections, the crossover effect in the columns of the various figures shows that they are not incidental.

The observations clearly showed that the PE teachers teaching style in the ordinary PE lessons and the teacher-directed PE, turned out to be approximately the same during the periods with teacher-directed PE and ordinary PE. This was because the PE teacher used a teacher-directed PE style in his ordinary teaching. In both periods (ordinary PE and teacher-directed PE), the PE teacher did not give the pupils any possibility to make choices. Because of these findings, the discussion will be between the self-determined PE (autonomy support) PE style, and the ordinary PE teacher style and a teacher-directed PE style together. A distinction between an ordinary PE style and a teacher-directed PE style do not seem to be meaningful considering our findings.

The results of our study are supported by those of other intervention studies indicating that self-determined instruction gives positive outcomes in pupils' happiness, mastery, well-being, contentment and level of activity (Prusak et al., 2004; Ward et al., 2008; Lonsdale et al., 2009, 2013; How et al., 2013), without having specifically studied these correlations. Also other studies point toward high levels of autonomy as preferred for the optimal pattern of outcomes (Van den Berghe et al., 2013, 2015; De Meyer et al., 2016; Haerens et al., 2018). Our finding is problematic according to a study of Haerens et al. (2013). In a study among PE teachers they found that the items that were part of the autonomy-supportive factor, were the least frequently observed in their study. The PE teachers in their study were rarely providing choice to their pupils, and hardly provided opportunities to practice independently. On the other side, several studies have shown that interventions have been successful in increasing autonomy support among teachers (Tessier et al., 2010; Cheon et al., 2012; Aelterman et al., 2014; Ulstad et al., 2018). Our findings will be discussed further in relation to the study's five independent variables.

### Self-Determination and Happiness

The analyses show that one of the intervention groups experienced significantly more happiness in the time when they were given more self-determination, compared to those periods when they had ordinary of teacher-directed PE. The hypothesis that increased self-determination in PE serves to increase pupils' experience of happiness is therefore fulfilled to a certain extent. The findings suggest that by being able to choose those activities that they most enjoy doing, the pupils' find PE more enjoyable and associated with happiness. This is in line with self-determination theory (Deci and Ryan, 2000). The results of Dismore and Bailey (2009) study show that the experience of fun was a critical factor for the creation of happiness in PE, and also Reid (1997) emphasizes the significance of values associated with happiness, as an important factor in the facilitation of PE. Wright (2004) points out that a particular activity places its own demands on the skills needed to accomplish it, but that, at the same time, there was the question of whether it included an element of pleasure. Kretchmar (1994) points out that the happiness one finds in an activity is an inner value. Such an inner value is closely associated with inner motivation. If pupils feel that an activity has an inner value for them, this can lead to increased inner motivation–a point also made by Deci and Ryan (2000).

### Self-Determination and Mastery

The analysis shows that although both intervention groups experienced increased mastery in the period with self-determination, compared to the periods with ordinary or teacher-directed PE, these differences were not significant. These results were significant before the Bonferroni corrections, but are no longer significant after (strict) Bonferroni corrections, where the p level for the group differences were near a borderline level (*p* = 0.070). Also the crossover effect we see in the columns belonging to the two intervention groups indicate that increased self-determination can have a positive effect on mastery, and shows a trend even though the differences are not significant in this study. Ulstad et al. (2018), found that motivation derived from autonomy is directly related to perceived competence. When pupils chose activities themselves, it was therefore reasonable to suppose that their inner motivation was stronger than in ordinary or teacher-directed instruction.

The observation notes show that when the pupils chose activities themselves, they chose many activities that the teacher ordinarily would not have included in his teaching (dance, taekwondo, cheerleading, ju-jitsu, climbing). Basketball, handball, football, bandy, strength training, stair training (suppleness), gymnastics, movement training, skipping, volleyball, injury prevention and dodgeball were other activities chosen. One can suppose, following the self-determination theory (Deci and Ryan, 1985, 2000; Ryan and Deci, 2007) and the findings of (Lagestad, 2017), that the pupils chose activities that they felt themselves to be competent in, leading to a greater sense of mastery. We suggest that when pupils themselves choose the activities, they are better able to reveal new aspects of themselves, and competencies the teacher maybe was not aware of. As well as providing the teacher with a wider scope for making an evaluation (something that the teacher pointed to during the observation), earlier research emphasizes the importance of pupils being able to demonstrate in PE activities that they have properly mastered (Lagestad, 2017; Lyngstad et al., 2019; Mikalsen and Lagestad, 2019).

### Self-Determination and Well-Being

The analysis showed that one of the intervention groups experienced significantly higher well-being in the period with self-determination, compared to the periods with ordinary and teacher-directed PE. Although the other intervention group experienced the greatest well-being in the period with self-determination, compared to the periods with ordinary and teacher-directed PE, the difference was not significant, and can be seen as incidental. The crossover effect we see in the columns relating to the two intervention groups does however indicate that the difference is not incidental. The hypothesis that increased self-determination raises the level of pupils' well-being is therefore fulfilled to a certain extent. This is in line with the standpoint of the study (Deci and Ryan, 2000), where self-determination theory emphasizes that increased self-determination facilitates well-being through increased internal motivation. This is also in agreement with several previous studies, as Ward et al. (2008), who contend that a greater experience of autonomy will positively affect pupils' well-being, and the findings of Ntoumanis (2005), who suggest that supporting pupils' autonomy, will positively impact pupils' well-being. Furthermore, the findings is somehow supported by a study of (Lagestad, 2017), who indicate that autonomy can positively affect pupils' well-being. This increased well-being resulting from increased self-determination can also be explained by Csikszentmihalyi (1990) “flow theory,” which he uses to explain well-being.

### Self-Determination and Contentment

The analyses show that in the periods where the intervention groups were able to choose activities themselves, they experienced a greater degree of contentment in PE, compared to the periods with ordinary and teacher-directed PE. Meanwhile, the control group also showed that they experienced significant differences in two of the periods. The crossover effect we see in the columns belonging to the two intervention groups, shows that the significance self-determination has for contentment, is not incidental. The results is somehow supported by (Lagestad, 2017). It is reasonable to suggest that the increased experience of self-determination goes together with autonomy, since the meeting of psychological needs is necessary in order to maintain and promote inner motivation (Ryan and Deci, 2007). When the pupils had self-determined PE, they had the opportunity to practice their chosen activities over a period of time–which was confirmed by the observations. Through such an approach the pupils were able to go into them in depth, by, for example, acquiring a difficult technique. This is in accordance with what Kretchmar (1994) claims contributes to a long-lasting contentment.

### Self-Determination and Activity Level

The analysis shows that in the periods where the intervention groups were able to choose activities themselves, they had significantly higher levels of MVPA compared to the periods of ordinary or teacher-directed PE. The control group showed up to also have significantly different activity levels between the two periods. However, the crossover effect we see in the columns belonging to the two intervention groups suggests that the effect self-determination has on activity level, is not incidental. Our results in this area are in conformity with many other earlier studies, showing that when pupils were able to choose for themselves, their PA level increased (Haerens et al., 2010; How et al., 2013; Lonsdale et al., 2013). On the other hand, Ward et al. (2008) found no connection between self-determined motivation and the level of activity. A study by Janssen and Leblanc (2010) recommended that children and youth 5–17 years of age should accumulate an average of at least 60 min per day and up to several hours of at least moderate intensity PA. Some of the health benefits can be achieved through an average of 30 min per day. More vigorous intensity activities should be incorporated or added when possible, including activities that strengthen muscle and bone. Our study showed that when the intervention groups chose activities themselves, they had significantly higher levels of MVPA compared to the periods of ordinary or teacher-directed PE. Our findings contribute to the knowledge of how to increase children and adolescents‘ daily activity level. Sigvartsen et al. (2016) demonstrated that by making use of interest-based PE, one profits in the form of health-related gains in life quality. In accordance with studies conducted by Lonsdale et al. (2013) and How et al. (2013), and from our theoretical standpoint in self-determination theory (Deci and Ryan, 1985, 2000; Ryan and Deci, 2007), we will argue that the significantly higher level of activity (in the form of both MVPA and CPM) in the period of self-determination, can be linked to the increased experience of self-determination (autonomy). On the other hand–the control group had also a significantly higher MVPA and CPM in one period than the other. The observation data suggest that this had a clear connection to the higher intensity activities chosen in the control group by the teacher in period 2, compared to period 1. The observation data showed that when the teacher held ordinary or teacher-directed physical classes, the teacher used a relatively large amount of time on: attendance procedures in the gymnasium, instructions, verbal instruction throughout the lesson, ending the lesson and so on–periods of time in which the pupils were passive. The teacher used a lot of time to talk, which can have contributed to the lower level of activity in the ordinary and teacher-directed instruction. When pupils chose the activities themselves, they were more quickly underway with the activity than when the teacher was directing the activity. This is in line with Patterson and Van Der Mars (2008), finding that pupils who were in close interaction with the teacher had the same level of activity as before, whilst those whose instruction took place distanced from the teacher, increased theirs. The PA level in our study are approximately the same as in other studies (Meyer et al., 2013; Chen et al., 2014; Andersen, 2017; Mayorga-Vega et al., 2017). Several of the activities that the teacher chose in teacher-directed PE and ordinary PE, was the same activities that the pupils chose in the self-determined PE. A lot of these activities do not promote to moderate and high intensity (Fairclough and Stratton, 2005b). This suggest that it‘s the intervention and the self-determination strategy that increases the level of activity, and not change of activities in the different periods. Kalajas-Tilga et al. (2020) found out that to enhance adolescents' MVPA in daily basis, the special focus should be put on increasing their intrinsic motivation toward physical education. When the adolescents‘ chose activity themselves, both MVPA and CPM were significantly higher than at ordinary and teacher-directed PE. By giving the adolescents‘ self-organized PE the focus was to increase their intrinsic motivation in PE. Delextrat et al. (2020) concluded that activity type could be associated with the intensity of PA in PE. By giving the adolescents‘ self-organized PE the purpose was to see if MVPA increased or went down in relation with the activity itself. When the adolescent chose activities themselves there was significantly higher levels of MVPA compared to the periods of ordinary or teacher-directed PE.

### Strengths and Limitations of the Study

A randomized selection of control group and intervention groups was made, and the same PE teacher was involved with all groups in all periods. Furthermore, their levels of PA were measured using accelerometers. This is seen as the best way in which to gather activity data about young people (Kolle et al., 2012), even though some activities, such as cycling, are underestimated. It should be noted here, however, that with the exception of one pupil who cycled in one PE lesson, none of the pupils cycled in the intervention period. The level of activity was measured as MVPA and CPM, which are standardized and highly regarded measures of activity level internationally. Another strength of the study is that the same researcher was present in all 24 of the lessons during which measurements were made. In this way the study's protocol was accurately followed, as well as data about each variable being gathered in order to support the data material from the accelerometer measurements and from the questionnaire–a strategy that strengthens the study's validity and reliability. The use of a crossover design also adds to the credibility of the study's findings.

The study is not without limitations. It is striking that the control group experienced a generally lower level of perception of having choice, happiness, well-being and contentment than the intervention groups at baseline. According to both the teacher and the observations, the control group differentiated itself by being less positive to PE than the other classes. This is supported by the statistical analysis which showed that the control group experienced significantly lower happiness, well-being and contentment in PE. In this way, one can imagine that the effect could have been greater if this class had been selected as an intervention class. This because the effect of having choices might have affected the control group in a more positive way more than the intervention groups, because of their lower baseline levels. Although the concepts of happiness, mastery, well-being and contentment have been formed on the basis of theory and factor analysis, these concepts have not been used and validated in previous research, and the validity of the measurements can be questioned. On the other hand, these four variables are constructed on the basis of logical and theoretical reasoning, where most of the questions in the items have high face validity, and are related according to the factor analyses. Furthermore, according to contentment and activity level, there were significant changes across time in the control group, that seems to indicate that natural fluctuations occur in some of the variables. A cautious interpretation of the findings is therefore needed. The observations showed that the PE teachers teaching style in the ordinary PE lessons and the teacher-directed PE, turned out to be approximately the same during the periods with teacher-directed PE and ordinary PE–and the importance of involving ordinary PE was limited in our study. Finally, we must highlight that past research has shown that perceived choice is only one facet of autonomy. Autonomy support, sometimes termed as “need support,” is a lot more than only providing additional choice. Future articles should include research which has adopted dimensions of autonomy support as cognitive, organizational and procedural in the context of physical education.

## Conclusion

Several studies have looked at self-determination, but none of them have looked specifically at self-determination in the context of happiness, mastery, well-being, contentment, and level of activity. The results from this study suggest that increased self-determination in PE positively affects young people's happiness, well-being, contentment, and level of activity. Even though the pupils' experience of mastery did not increase significantly, the findings here suggest that one cannot assert that self-determination does not affect pupils' mastery in PE. However, it is important to highlight that some natural fluctuations occur in some of the variables in the control group, especially related to PA level, and a cautious interpretation of this finding is therefore needed. Taking this into mind, the findings of this study suggest that it is important that pupils are able to make their own choices in PE, and that PE teacher's practice ought to include periods in which the pupils are able to make their own choices regarding self-organized activities. As suggested by Haerens et al. (2013), the results also suggest the need for PE teacher education programs and continuous professional development programs to include a module on how to teach in an autonomy-supportive way. Further research should be based on intervention studies studying self-determination over a longer continuous period, in classes with both older and younger pupils, but also where self-determination does not necessarily take as its starting point the choice of activities during PE lessons.

## Data Availability Statement

The raw data supporting the conclusions of this article will be made available by the authors, without undue reservation.

## Ethics Statement

The studies involving human participants were reviewed and approved by Norwegian Centre for Research Data (NSD). Written informed consent to participate in this study was provided by the participants' legal guardian/next of kin.

## Author Contributions

All authors listed have made a substantial, direct and intellectual contribution to the work, and approved it for publication.

## Conflict of Interest

The authors declare that the research was conducted in the absence of any commercial or financial relationships that could be construed as a potential conflict of interest.
